# Sudden Unexpected Infant Death Rates and Social Determinants of Health Among Hispanic Infants

**DOI:** 10.1001/jamanetworkopen.2025.15986

**Published:** 2025-06-16

**Authors:** Bianca Quiñones-Pérez, Christopher Cortina, Anja Sandholm, Kathryn P. Gray, Richard D. Goldstein

**Affiliations:** 1 Division of General Pediatrics, Boston Children’s Hospital, Boston, Massachusetts; 2 Department of Pediatrics, Harvard Medical School, Boston, Massachusetts; 3 Biostatistics and Research Design Center, Boston Children’s Hospital, Boston, Massachusetts; 4 Robert’s Program on Sudden Unexpected Death in Pediatrics, Boston Children’s Hospital, Boston, Massachusetts

## Abstract

**Importance:**

Sudden unexpected infant death (SUID) is the leading cause of postneonatal mortality, with disparities attributed to social determinants of health (SDOH). SUID in the Hispanic population has received limited attention, despite the fact that one-fourth of US children are Hispanic.

**Objective:**

To compare SUID rates and risk factors among Hispanic and non-Hispanic infants, and associated interactions among SUID, SDOH, and acculturation.

**Design, Setting, and Participants:**

This US nationwide retrospective cohort study used US National Center for Health Statistics (NCHS) linked birth and infant death data, and Pregnancy Risk Assessment Monitoring System (PRAMS) data from 1996 to 2017. All live births (NCHS) or participants (PRAMS) with documented maternal ethnicity were included. Data were analyzed from February to October 2024.

**Exposure:**

Maternal Hispanic ethnicity.

**Main Outcomes and Measures:**

The primary outcome was postneonatal SUID occurring at age 28 to 364 days, as designated by *International Classification of Diseases, Ninth Revision* codes 798, 799, and 913 (1996-1999), and *International Statistical Classification of Diseases and Related Health Problems, Tenth Revision* codes R95, R99, and W75 (2000-2017). Odds ratios (ORs) were calculated by exposure, and adjusted ORs controlled for risk factors in a multivariable model. Maternal nativity variables associated with race, poverty, local SUID rates, and region of origin were investigated. Subgroup analysis explored the relative association of Hispanic ethnicity with SUID risk factors. Maternally reported risk factors were compared according to Hispanic ethnicity.

**Results:**

Among 88 067 608 live births (median [IQR] maternal age, 27 [22-32] years; median [IQR] gestational age, 39 [38-40] weeks) and 54 828 SUID deaths, there were 7173 SUID deaths among 19 887 156 Hispanic infants. The SUID rate was lower for Hispanic infants (0.36 deaths per 1000 live births) than for non-Hispanic infants (0.70 deaths per 1000 live births), across essentially all factors analyzed. Overall, Hispanic infants had 33% lower odds of SUID than non-Hispanic infants (adjusted OR, 0.67; 95% CI, 0.65-0.69). Infants of non–US-born Hispanic mothers had lower SUID rates, regardless of race, county-level poverty, local SUID rates, or Hispanic region of origin, except Puerto Rican infants. Altered associations with detrimental and protective factors were found, despite a mixed picture of risk.

**Conclusions and Relevance:**

In this cohort study of SUID in infants born from 1996 to 2017, Hispanic infants had lower SUID rates than non-Hispanic infants, despite adverse SDOH. Risk factors operated differently in Hispanic infants, challenging current conceptualizations of risk. Understanding how risk operates in Hispanic populations can help to better address the mortality burden of SUID.

## Key Points

**Question:**

What are the rates of sudden unexpected infant death (SUID) among Hispanic infants, and how are risk factors and social determinants of health associated with SUID rates within the population?

**Findings:**

In this cohort study analyzing 54 828 SUID deaths over 21 years of US postneonatal mortality data, SUID rates were lower, and risk factors and social determinants had different associations with risk among Hispanic infants vs non-Hispanic infants. Infants of nonimmigrant Hispanic mothers had higher SUID risk.

**Meaning:**

Understanding the risk variables and social determinants of health among Hispanic infants may have implications across all ethnic groups and help to better understand and address SUID mortality.

## Teaser

This cohort study examines rates of sudden unexpected infant death and associated interactions among social determinants of health (SDOH) in Hispanic infants.

## Introduction

Sudden infant death syndrome (SIDS) is the leading cause of postneonatal mortality in the US.^1^ SIDS surveillance is complicated by variations in diagnostic practices among death certifiers, where unexplained infant deaths with often indistinguishable circumstantial and clinical details are not reliably classified.^2,3^ This has necessitated the development of a special cause-of-death category, designated sudden unexpected infant death (SUID), to incorporate infant deaths certified as SIDS, other ill-defined or unspecified causes of death (undetermined), and accidental suffocation and strangulation in bed.^4^ Our understanding of the problem’s causes continues to evolve, yet consensus holds that it involves an interplay between external factors and intrinsic vulnerabilities in affected infants.^5,6^ Like most health outcomes, disparities typically attributed to social determinants of health (SDOH) are seen in SUID rates among US racial and ethnic subgroups.^5,7^

Since 1996, the National Center for Health Statistics (NCHS) has collected data categorizing individuals who self-identify as Hispanic. Although frequently presented as a discrete demographic category, Hispanic individuals represent a group with heterogeneous races, heritages, nationalities, lineages, countries of birth, and countries of parental or ancestral origin. Their nativity may be US-born or non–US-born, and their ancestry may trace to Latin America or Spanish-speaking cultures, regardless of race. Individuals born in Puerto Rico are US citizens who are considered non–US-born in US census data.

Hispanics make up the so-called majority minority in the US. According to the 2020 census, 25.7% (18.8 million) of children younger than 18 years in the US are Hispanic.^8^ Over 30% of non–US-born Hispanic children live in poverty, nearly twice the national average, as do children with 2 Hispanic parents, although poverty rates decrease in subsequent US-born generations.^9^ Only 23% of non–US-born Hispanic immigrants are fluent in English, which affects health literacy and health care access.^10,11^ Despite these apparently adverse SDOH, the Hispanic infant mortality rate is comparable to that of non-Hispanic White infants. This is held as an example of the so-called Hispanic paradox,^12,13^ whereby Hispanic health outcomes exceed mortality and morbidities otherwise associated with SDOH.^14,15^

Understanding racial and ethnic differences in child health is vital to reducing disparities^16^ and may also yield important insights into the mechanisms of risk. Despite the importance of the Hispanic pediatric population, their SUID health outcomes have received little comprehensive attention.^17,18,19^ This research was conducted to compare SUID rates in infants born to Hispanic and non-Hispanic mothers (hereafter, Hispanic and non-Hispanic infants, respectively), and to explore the associations among SUID rates, risk factors, SDOH, and acculturation factors in the Hispanic population.

## Methods

### Data Sources

This national retrospective cohort study used linked birth and infant death records from the NCHS from 1996 to 2017 to include the most current available data combining 1 year of birth records with 2 years of death reporting.^20^ Data were weighted as recommended by NCHS to adjust for approximately 2% to 3% of deaths that cannot be linked to their corresponding birth certificates. US Census data were used for county (2020) and poverty (2021) demographics.

We used contemporaneous (1996-2017) data from the Pregnancy Risk Assessment Monitoring System (PRAMS),^21^ a population-based surveillance system of self-reported behaviors and experiences before, during, and after pregnancy among people with a recent live birth, to evaluate SUID-associated risk as reported by Hispanic and non-Hispanic mothers. PRAMS cosleeping data were only available for 1996 to 2008. PRAMS data were weighted to adjust for sampling design, noncoverage, and nonresponse to represent the entire population of births in a given state or jurisdiction. We followed Strengthening the Reporting of Observational Studies in Epidemiology (STROBE) reporting guidelines in preparing this manuscript. Data sources are deidentified and publicly available, exempting the study from institutional board review and the need for informed consent, in accordance with 45 CFR §46.104.

### Study Cohorts, Exposures, Confounders, and Outcome

**Figure 1.**
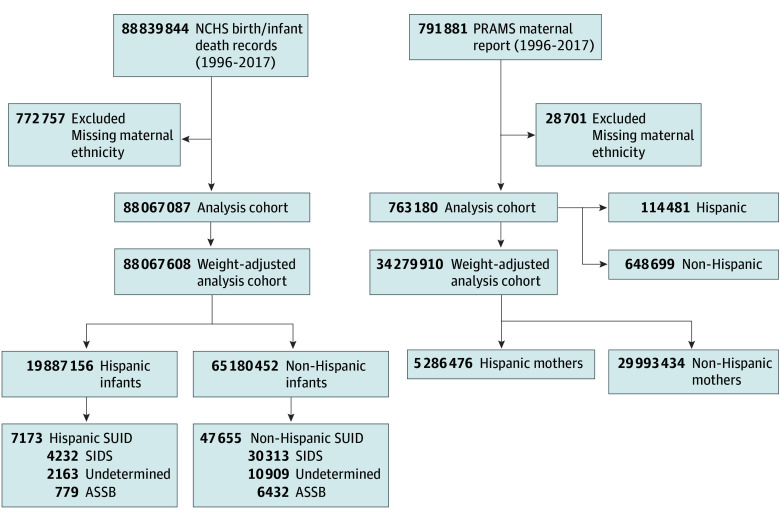
Case Flow Information From National Center for Health Statistics (NCHS) and Pregnancy Risk Assessment Monitoring System (PRAMS) Datasets Flowchart shows participant enrollment. Sudden infant death syndrome (SIDS) is designated with *International Classification of Diseases, Ninth Revision (ICD-9)* code 798 and *International Statistical Classification of Diseases and Related Health Problems, Tenth Revision (ICD-10)* code R95, including all subclassifications. Undetermined refers to ill-defined and unknown causes and is designated with *ICD-9* code 799 and *ICD-10* code R99, including all subclassifications. Accidental suffocation and strangulation in bed (ASSB) is designated with *ICD-9 *code 913 and *ICD-10 *code W75, including all subclassifications. SUID indicates sudden unexpected infant death.

Inclusion criteria included all live-born infants with maternal ethnicity documented on their birth certificates (NCHS) and participants with documented ethnicity (PRAMS) (Figure 1). The primary exposure was maternal Hispanic ethnicity, categorized as Hispanic or Latino (hereafter, Hispanic) or non-Hispanic or non-Latino (hereafter, non-Hispanic). Using NCHS data, we assessed maternal SUID risk factors (race, age, marital status, education, cigarette use, and prenatal care), infant SUID risk factors (gestational age, birth weight, and sex), and SDOH (poverty, rurality, maternal nativity, and maternal region of origin) as potential confounders. Descriptive paternal data were also included. Definitions for variables can be found in eTable 1 in Supplement 1.

The primary outcome, postneonatal SUID, incorporated *International Classification of Diseases, Ninth Revision (ICD-9)* codes 798, 799, and 913 (1996 to 1999), and *International Statistical Classification of Diseases and Related Health Problems, Tenth Revision (ICD-10)* codes R95, R99, and W75 (2000 to 2017), including all subclassifications, occurring at 28 to 364 days of age. PRAMS data were used to investigate population-level SUID risk factors of prone sleeping, cosleeping with mother, maternal smoking (never vs any), maternal age under 20 years, unmarried mother, mother with no high school degree, late or absent prenatal care, inadequate or intermediate Kotelchuck index,^22^ premature birth (<37 weeks), low birth weight (<2500 g), and breastfeeding.

### Statistical Analysis

We used descriptive statistics to summarize maternal SUID risk factors, infant SUID risk factors, and SDOH according to maternal Hispanic vs non-Hispanic ethnicity. SUID rates were calculated as the number of postneonatal SUID deaths per 1000 live births (1996-2017) overall and within each category.

We used weighted logistic regression models to examine the association between SUID and maternal ethnicity, estimating the odds ratio (OR) for SUID comparing Hispanic with non-Hispanic infants (reference group). A multivariable model was performed to report the adjusted SUID odds ratio (aOR), controlling for maternal race, late or absent prenatal care, cigarette use, marriage status, county-level poverty, rurality, and birth year.

Within the Hispanic infant population, we calculated SUID rates by race, county-level poverty, local SUID rates, and Hispanic region of origin, stratified by maternal nativity (US-born vs non–US-born). A local estimated scatterplot smoothing plot was used to display comparative SUID rates over time applying NCHS weighting.

We performed weighted logistic regression to examine the association between SUID and maternal Hispanic ethnicity for select risk factor subgroups available in NCHS data. We estimated ORs for SUID comparing Hispanic with non-Hispanic infants (reference group) in the subgroups of maternal race, nativity, prenatal care, cigarette use, marriage status, poverty, rurality, and birth year.

We summarized distributions of available SUID risk factors from weighted PRAMS data according to maternal Hispanic ethnicity and calculated ORs for the presence of SUID risk factors (yes or no) comparing Hispanic with non-Hispanic mothers using weighted logistic regression models. Records with unknown or missing values were categorized as unknown and were included in all regression model analyses. ORs are reported with 95% CIs; statistical significance is defined as a 95% CI that does not include the reference value of 1. SAS statistical software version 9.4 (SAS Institute) was used for dataset analysis and statistical modeling, and R statistical software version 4.3.2 (R Project for Statistical Computing) was used for generating local estimated scatterplot smoothing plots. Data were analyzed from February to October 2024.

## Results

### SUID Rates in Hispanic vs Non-Hispanic Infants

Our 21-year (1996-2017) cohort included 88 067 608 live births (median [IQR] maternal age, 27 [22-32] years; median [IQR] gestational age, 39 [38-40] weeks) and 54 828 SUID deaths. Of those, 19 887 156 infants (22.6%) were Hispanic, accounting for 7173 SUID deaths (0.36 deaths per 1000 live births). Hispanic infants had lower rates of SUID compared with non-Hispanic infants in all racial groups (0.70 deaths per 1000 live births), except Asian and Pacific Islander infants (Table 1). SUID rates in Hispanic infants were lower in essentially every category evaluated, except compared with infants of non–US-born mothers, where they were roughly equivalent. Paternal data similarly showed lower rates among infants of Hispanic fathers across risk factors, but missing data undermine reliability (eTable 2 in Supplement 1). When analyzed by race, Hispanic Black and Hispanic White infants had similar SUID rates, which were both lower than rates among non-Hispanic Black and non-Hispanic White infants, respectively. SUID deaths in Black Hispanic infants occurred at one-quarter the rate of non-Hispanic Black infants, (0.21 vs 0.87 deaths per 1000 births), but Black Hispanic infants experienced a SUID rate exceeding that of any other race among Hispanic infants.

**Table 1.** Counts and SUID Rates From 1996 to 2017 for Maternal and Infant SUID Risk Factors and Social Determinants According to Maternal Hispanic vs Non-Hispanic Ethnicity^a^

Characteristic	Total participants, No. (%) (N = 88 067 608)	Maternal Hispanic ethnicity^b^			Maternal non-Hispanic ethnicity		
		Participants, No. (%)		SUID rate (0.36)^c^	Participants, No. (%)		SUID rate (0.70)^c^
		No SUID (n = 19 879 983)	SUID (n = 7173)		No SUID (n = 68 132 797)	SUID (n = 47 655)	
Maternal SUID risk factors							
Maternal race							
American Indian and Alaska Native	930 273 (1.1)	102 453 (0.5)	40 (0.6)	0.39	826 933 (1.2)	847 (1.8)	1.02
Asian and Pacific Islander	5 035 306 (5.7)	179 843 (0.9)	28 (0.4)	0.15	4 854 799 (7.1)	636 (1.3)	0.13
Black	13 619 513 (15.5)	740 619 (3.7)	155 (2.2)	0.21	12 867 482 (18.9)	11 257 (23.6)	0.87
White	67 730 248 (76.9)	18 736 040 (94.2)	4039 (56.3)	0.22	48 970 877 (71.9)	19 292 (40.5)	0.39
More than one race	377 559 (0.4)	60 931 (0.3)	0	NA	316 628 (0.5)	0	NA
Unknown	374 710 (0.4)	60 097 (0.3)	2912 (40.6)	NA	296 078 (0.4)	15 623 (32.8)	NA
Maternal age, y							
<20	8 485 043 (9.6)	2 634 085 (13.2)	1878 (26.2)	0.71	5 839 387 (8.6)	9693 (20.3)	1.66
20-24	21 092 767 (24.0)	5 594 592 (28.1)	2711 (37.8)	0.48	15 476 882 (22.7)	18 582 (39.0)	1.2
25-29	24 506 100 (27.8)	5 389 035 (27.1)	1542 (21.5)	0.29	19 104 820 (28.0)	10 704 (22.5)	0.56
30-34	21 321 221 (24.2)	3 910 641 (19.7)	690 (9.6)	0.18	17 404 169 (25.5)	5720 (12.0)	0.33
35-39	10 340 925 (11.7)	1 904 841 (9.6)	289 (4.0)	0.15	8 433 306 (12.4)	2489 (5.2)	0.3
≥40	2 321 552 (2.6)	446 789 (2.2)	63 (0.9)	0.14	1 874 233 (2.8)	467 (1.0)	0.25
Maternal marital status							
Married	54 870 267 (62.3)	10 101 508 (50.8)	2471 (34.4)	0.24	44 748 901 (65.7)	17 387 (36.5)	0.39
Single	32 738 193 (37.2)	9 557 238 (48.1)	4702 (65.6)	0.49	23 145 985 (34.0)	30 268 (63.5)	1.31
Unknown	459 148 (0.5)	221 237 (1.1)	0	NA	237 911 (0.3)	0	NA
Maternal education^d^							
High school degree or greater	13 216 369 (15.0)	6 471 572 (32.6)	2897 (40.4)	0.45	6 729 903 (9.9)	11 997 (25.2)	1.78
Did not achieve high school degree	54 697 062 (62.1)	9 093 400 (45.7)	2652 (37.0)	0.29	45 577 940 (66.9)	23 070 (48.4)	0.51
Unknown	20 154 177 (22.9)	4 315 011 (21.7)	1624 (22.6)	NA	15 824 954 (23.2)	12 588 (26.4)	NA
Maternal cigarette use							
Yes	7 418 133 (8.4)	374 161 (1.9)	662 (9.2)	1.77	7 028 623 (10.3)	14 687 (30.8)	2.09
No	68 713 227 (78.0)	15 500 926 (78.0)	4929 (68.7)	0.32	53 181 235 (78.1)	26 137 (54.8)	0.49
Unknown	11 936 248 (13.6)	4 004 896 (20.1)	1581 (22.0)	NA	7 922 939 (11.6)	6832 (14.3)	NA
Late or absent prenatal care^e^							
Yes	4 158 254 (4.72)	1 356 654 (6.8)	846 (11.4)	0.62	2 796 121 (4.1)	4633 (9.4)	1.65
No	79 661 355 (90.5)	17 548 446 (88.3)	6144 (83.0)	0.35	62 065 011 (91.1)	41 755 (9.4)	0.67
Unknown	4 248 005 (4.82)	974 656 (4.9)	411 (5.5)	NA	3 269 975 (4.4)	2882 (6.0)	NA
Infant SUID risk factors							
Infant’s gestational age							
Preterm	10 406 216 (11.8)	2 286 340 (11.5)	1619 (22.6)	0.71	8 107 035 (11.9)	11 221 (23.5)	1.38
Full term	77 206 360 (87.7)	17 394 570 (87.5)	5361 (74.7)	0.31	59 770 459 (87.7)	35 971 (75.4)	0.60
Unknown	455 032 (0.52)	199 069 (1.0)	197 (2.75)	NA	255 270 (0.37)	496 (1.04)	NA
Infant’s birth weight							
Very low	1 283 201 (1.46)	238 873 (1.20)	281 (3.8)	1.17	,041 895 (1.5)	2152 (4.4)	2.06
Low	5 734 036 (6.5)	1 112 910 (5.6)	1058 (14.3)	0.95	4 601 885 (6.8)	8.182 (16.6)	1.77
Appropriate	81 028 247 (92.0)	18 514 756 (93.1)	6060 (81.9)	0.33	62 468 435 (91.7)	38 996 (79.0)	0.62
Unknown	22 130 (0.03)	3219 (0.02)	2 (0.03)	NA	18 892 (0.03)	19 (0.04)	NA
Infant’s sex							
Female	43 002 016 (48.8)	9 739 412 (49.0)	2906 (40.5)	0.3	33 240 447 (48.8)	19 252 (40.4)	0.58
Male	45 065 592 (51.2)	10 140 571 (51.0)	4267 (59.5)	0.42	34 892 350 (51.2)	28 403 (59.6)	0.81
Other social determinants							
County-level poverty^f^							
High poverty	6 746 102 (7.7)	1 724 899 (8.7)	700 (9.8)	0.41	5 015 113 (7.4)	5390 (11.3)	1.07
Low poverty	81 084 671 (92.1)	18 027 913 (90.7)	6444 (89.8)	0.36	63 008 157 (92.5)	42 157 (88.5)	0.67
Unknown	236 836 (0.3)	127 171 (0.6)	29 (0.4)	NA	109 527 (0.2)	109 (0.2)	NA
County rurality^g^							
Mostly or completely rural	6 651 922 (7.6)	553 059 (2.8)	265 (3.7)	0.48	6 092 763 (8.9)	5835 (12.2)	0.96
Mostly urban	81 178 850 (92.2)	19 199 753 (96.6)	6879 (95.9)	0.36	61 930 507 (90.9)	41 711 (87.5)	0.67
Unknown	236 836 (0.3)	127 171 (0.6)	29 (0.4)	NA	109 527 (0.2)	109 (0.2)	NA
Maternal nativity^h^							
US born	67 755 591 (76.9)	8 353 830 (42.0)	4652 (64.9)	0.56	59 351 587 (87.1)	45 522 (95.5)	0.77
Non–US born	20 066 961 (22.8)	11 487 883 (57.8)	2492 (34.7)	0.22	8 574 679 (12.6)	1907 (4.0)	0.22
Unknown	245 056 (0.3)	38 270 (0.2)	29 (0.4)	NA	206 531 (0.3)	226 (0.5)	NA
Maternal Hispanic region of origin^i^							
Non-Hispanic	68 180 452 (77.4)	NA	NA		68 132 797 (100.0)	47 655 (100.0)	0.7
Mexico	13 182 610 (15.0)	13 177 960 (66.3)	4650 (64.8)	0.35	NA	NA	NA
Puerto Rico	1 405 221 (1.6)	1 404 382 (7.1)	839 (11.7)	0.6	NA	NA	NA
Cuba	364 162 (0.4)	364 077 (1.8)	85 (1.2)	0.23	NA	NA	NA
Central or South America	2 939 758 (3.3)	2 939 114 (14.8)	644 (9.0)	0.22	NA	NA	NA
Other and unknown Hispanic	1 995 406 (2.3)	1 994 450 (10.0)	956 (13.3)	0.48	NA	NA	NA

Abbreviations: NA, not applicable; SUID, sudden unexpected infant death.

^a^ Data were obtained from the linked birth and infant death records from the National Center for Health Statistics.

^b^ Refers to mother’s self-reported race and ethnicity. All participants reporting Hispanic ethnicity were considered Hispanic; all participants not reporting Hispanic ethnicity were assigned to self-reported race.

^c^ Refers to postneonatal SUID rate per 1000 live births.

^d^ Did not achieve high school degree includes 0 to 11 years education (1996-2002) and 12th grade with no diploma or less (2003-2017). High school degree or greater includes 12 years or more education (1996-2002) and high school degree or greater (including some college, associate degree, bachelor’s degree, and graduate degree) (2003-2017).

^e^ Refers to first prenatal care appointment occurring after the 27th week of gestation, or not at all.

^f^ High poverty county is defined as more than 20% of the infant’s birth county population’s income below the poverty level in the US Census 2020.

^g^ Urban is defined as 50% or more of the infant’s birth county’s population living in an urban census tract in the US Census 2020.

^h^ For maternal place of birth, US-born pertains to mothers born in the continental US. Non–US-born mothers were born outside continental US, including US territories.

^i^ Refers to region of mother’s or mother’s family of origin, among Hispanic mothers.

The estimated odds of SUID were 48% lower in Hispanic infants compared with non-Hispanic infants (OR, 0.52; 95% CI, 0.50-0.53). When adjusted for SUID risk factors, Hispanic infants had 33% lower odds of SUID than non-Hispanic infants (aOR, 0.67; 95% CI, 0.65-0.69) (eTable 3 in Supplement 1).

### Hispanic SUID Rates Over Time Analyzed by Maternal Hispanic Nativity

**Figure 2.**
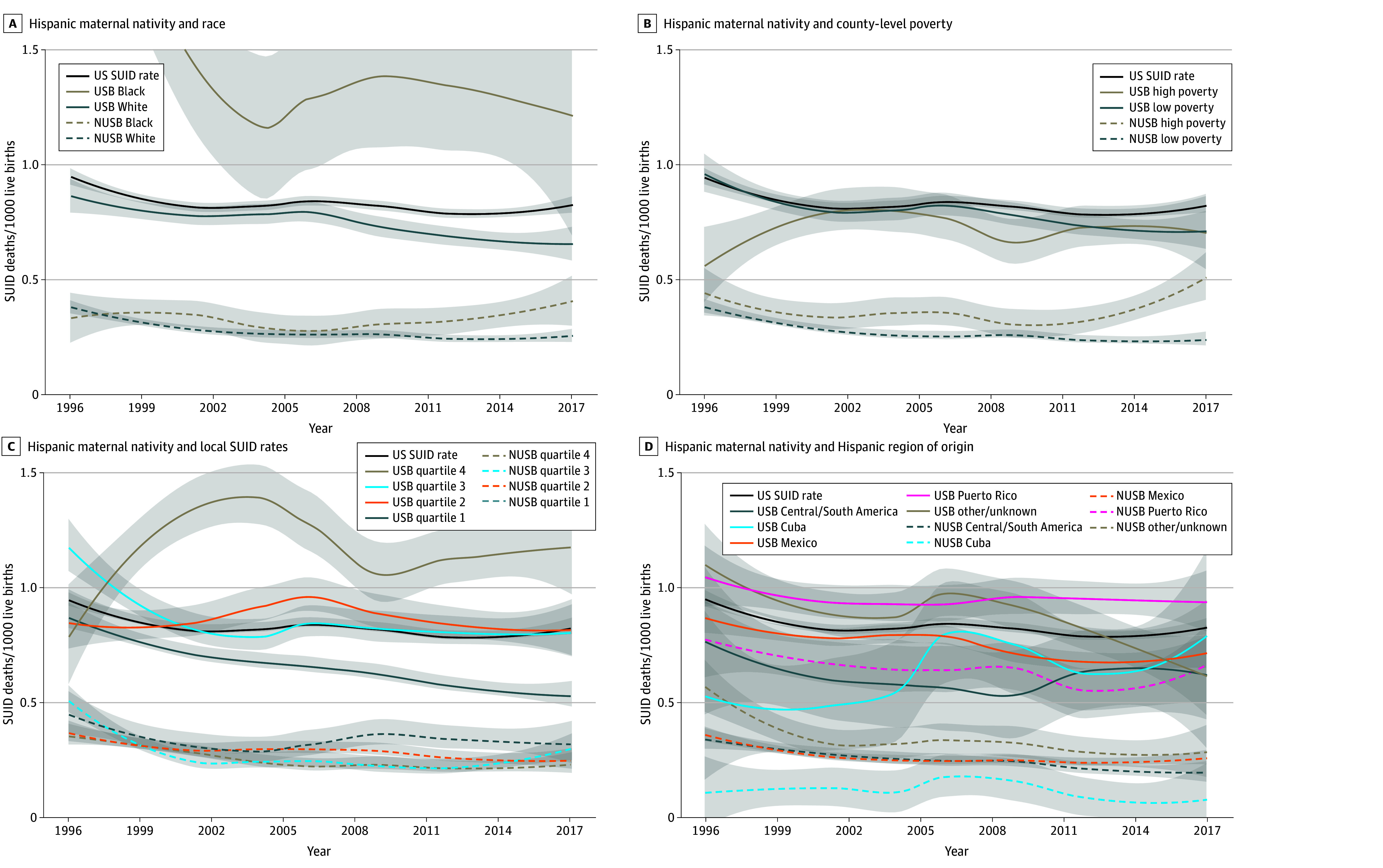
Sudden Unexpected Infant Death (SUID) Rates Over Time for Infants Born to Hispanic Mothers by Race, Poverty, Local SUID Rates, and Hispanic Regions of Origin Graphs show data for Hispanic maternal nativity and race (A), Hispanic maternal nativity and county-level poverty (B), Hispanic maternal nativity and local SUID rates, based on cumulative state SUID rates 1996 to 2017, matched based on state of birth (quartile 4 is the highest rate) (C), and Hispanic maternal nativity and Hispanic region of origin (D). NCHS data are presented as a local estimated scatterplot smoothing (span = 0.75, degree = 2) plot showing smoothed trends of SUID over time. The shaded area around the curves represents the 95% CI for the smoothed line. NUSB indicates non–US born; USB, US born.

Among Hispanic infants, those born to non–US-born Hispanic mothers had lower SUID rates than infants born to US-born Hispanic mothers, regardless of race, county-level poverty, local SUID rates, or region of origin (Figure 2). SUID rates for infants born to US-born Hispanic mothers were higher than those born to non–US-born Hispanic mothers and approached or surpassed the US SUID rate across all categories.

When stratified by race, infants born to non–US-born Hispanic Black and non–US-born Hispanic White mothers had similar rates, both lower than the overall US SUID rate (Figure 2A). Infants born to US-born Hispanic Black mothers had the highest rate among Hispanic racial subgroups, although it was lower than that of infants born to US-born non-Hispanic Black mothers after 1999 (eFigure in Supplement 1).

Nativity appears to be associated with SUID risk regardless of county-level poverty (Figure 2B). Infants born to non–US-born Hispanic mothers had similarly lowered rates compared with infants born to US-born Hispanic mothers in both high and low poverty counties, with SUID rates for infants born to US-born Hispanic mothers approaching the overall US SUID rate. SUID rates increased with each successive SUID rate quartile, as expected, yet SUID rates for infants born to non–US-born Hispanic mothers, regardless of community rates, were well below those for infants of US-born Hispanic mothers (Figure 2C).

Most Hispanic non–US-born maternal regions of origin were associated with lower SUID rates than the US average, except for infants born to non–US-born (islander) Puerto Rican mothers, who approached the US SUID rate. Infants born to Puerto Rican mothers, both US-born and non–US-born, had the highest rates of SUID compared with infants born to mothers of other Hispanic regions of origin within each nativity group (Figure 2D).

### Subgroup Analysis

**Figure 3.**
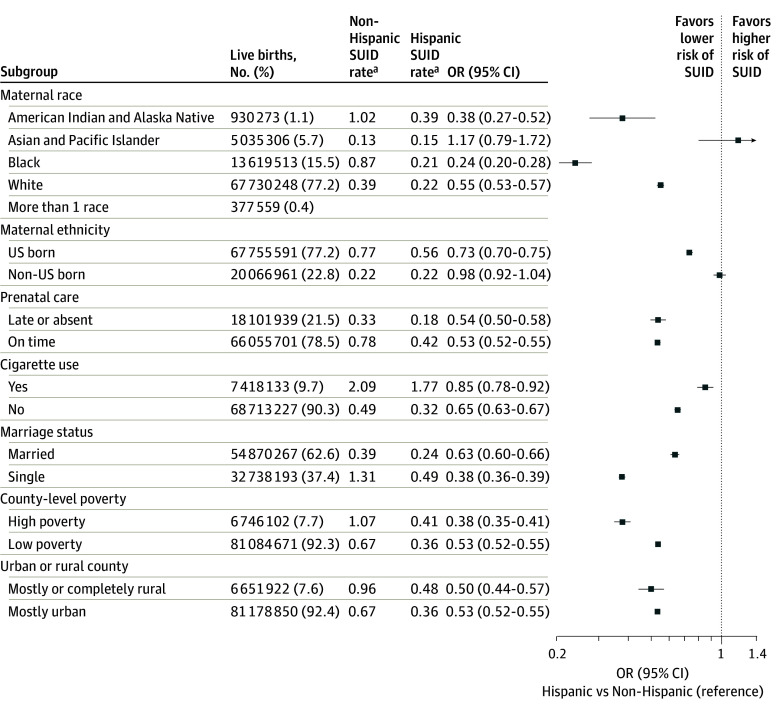
Association of Hispanic Maternal Ethnicity With Sudden Unexpected Infant Death (SUID) According to Risk Subgroups Plots shows the odds of SUID for Hispanic infants compared with non-Hispanic infants (reference) across different risk factor subgroups. Squares represent the odds ratio (OR) for each subgroup, and the width of the horizontal line shows the 95% CI. Individuals with missing data were not included in the analysis. ^a^Data are postneonatal SUID rates per 1000 live births over 21 years (1996-2017).

The odds of SUID were consistently lower (ORs <1) in Hispanic infants compared with non-Hispanic infants across most SUID risk factor subgroups. The only exceptions are Asian Pacific Islander infants, among whom Hispanic infants had a higher SUID OR, and infants born to non–US-born mothers, among whom Hispanic infants had the same odds as non-Hispanic infants. The degree in which the odds of SUID are reduced in Hispanic infants vary in each subgroup, with the most significant association shown for infants of Black mothers, for whom the odds of SUID were reduced by 76% (OR, 0.24; 95% CI, 0.20-0.28) for Black Hispanic infants compared with Black non-Hispanic infants (Figure 3). Infants born to Hispanic mothers who smoke had a 15% reduced risk of SUID compared with infants born to non-Hispanic mothers who smoke, while infants born to nonsmoking Hispanic mothers had an almost 40% comparative reduction in SUID risk. Infants of Hispanic mothers with late prenatal care had less than half the SUID risk of infants of non-Hispanic mothers.

### SUID Risk Factors

PRAMS data showed that Hispanic and non-Hispanic households had comparable rates of cosleeping, prematurity, and low birth weight. Hispanic mothers were more likely to be young, unmarried, less educated, and to have received late or absent prenatal care. In contrast, Hispanic mothers were less likely to place their infants prone to sleep, formula feed, or smoke cigarettes than non-Hispanic mothers, with the greatest difference seen in maternal smoking rates (Table 2).

**Table 2.** Hispanic Ethnicity and Maternal Self-Reported Risk Factors for SUID

SUID risk factors	Weighted % (95% CI)			Hispanic vs non-Hispanic, OR (95% CI)^a^
	Total (N = 763 180)	Hispanic mother (n = 114 481)	Non-Hispanic mother (n = 648 699)	
Any maternal smoking	10.6 (10.5-10.7)	3.0 (2.9-3.2)	12.0 (11.8-12.1)	0.23 (0.22-0.24)
Prone sleeping	14.0 (13.9-14.2)	10.7 (10.4-11.0)	14.6 (14.5-14.8)	0.70 (0.67-0.72)
Low birth weight	7.3 (7.2-7.3)	6.6 (6.5-6.7)	7.4 (7.4-7.5)	0.89 (0.87-0.91)
Premature birth	8.9 (8.8-9.0)	8.2 (8.0-8.4)	9.0 (8.9-9.1)	0.91 (0.88-0.93)
Infant sleeps with mother sometimes or always^b^	60.3 (59.1-61.4)	60.8 (58.0-63.5)	60.1 (58.9-61.3)	1.03 (0.91-1.17)
Late or absent prenatal care	1.7 (1.7-1.8)	2.2 (2.1-2.4)	1.6 (1.6-1.7)	1.38 (1.28-1.48)
Maternal age <20 y	9.0 (8.9-9.1)	11.6 (11.3-11.9)	8.5 (8.4-8.6)	1.41 (1.37-1.45)
Inadequate or intermediate Kotelchuck Index	24.4 (24.3-24.6)	31.8 (31.4-32.2)	23.1 (22.9-23.2)	1.55 (1.52-1.59)
Unmarried mother	37.0 (36.8-37.1)	50.4 (50.0-50.9)	34.5 (34.3-34.7)	1.93 (1.89-1.97)
Mother has no high school degree	16.8 (16.7-17.0)	41.3 (40.8-41.7)	12.4 (12.3-12.5)	4.96 (4.85-5.07)
Breastfeeding^c^	80.2 (80.0-80.4)	86.3 (85.9-86.8)	79.1 (78.8-79.3)	1.67 (1.60-1.75)

Abbreviations: OR, odds ratio; SUID, sudden unexpected infant death.

^a^ The odds of reporting a SUID risk factor comparing Hispanic mothers vs non-Hispanic mothers (reference).

^b^ Cosleeping data were available from 1996 to 2008.

^c^ Breastfeeding is considered a protective factor against SUID.

## Discussion

In this retrospective cohort study analyzing 21 years of US postneonatal mortality data, we found that SUID rates for Hispanic infants were significantly lower than those for non-Hispanic infants, as previously reported.^19,23^ SUID rates in Hispanic infants were lower than those for non-Hispanic infants in virtually every risk or demographic category evaluated, including rates associated with intrinsic risk factors for SUID such as prematurity and low birth weight. Infants born to non–US-born Hispanic mothers had markedly lower SUID rates regardless of race, poverty, local SUID rate, or Hispanic region of origin, except for infants born to Puerto Rican mothers. Hispanic mothers experienced high levels of socially mediated SUID risk factors, including younger age, less education, unmarried status, and inadequate prenatal care, whereas their infants benefited from less maternal smoking, more breastfeeding, and greater sleep placement in the supine position. However, even identified adverse risk factors showed a muted association with SUID outcomes among Hispanic infants in the subgroup analysis.

It is difficult to weigh the countervailing influences of risk factors for SUID in this population. Although Hispanic mothers report less placement of their infants in the prone position for sleep and less tobacco use, Hispanic mothers are younger, less educated, more likely to be unmarried, and more frequently receive inadequate prenatal care. Hispanic mothers cosleep with their infants at least as often as non-Hispanic mothers,^24^ and more than one-half of Hispanic SUID cases died in a shared sleep environment.^25^ Interestingly, both detrimental and protective risk factors for SUID operated differently in the Hispanic population: detrimental factors were less associated with SUID than in non-Hispanic infants, and protective associations were amplified. For example, infants born to Hispanic mothers who smoke had a 15% reduced risk of SUID compared with infants born to non-Hispanic mothers who smoke, while infants born to nonsmoking Hispanic mothers had an almost 40% comparative reduction in SUID risk. Infants of Hispanic mothers with late prenatal care had less than half the SUID risk of infants of non-Hispanic mothers. These results showcase differences in the impact of any given risk factor on SUID outcomes in the Hispanic population, defying existing research on SUID risk calculation^23,26,27^ and challenging the basic framework that risk factor modification uniformly affects outcomes.

It is important to recognize the pervasive influence of race on US SUID rates, which extends to Black Hispanic infants. Although SUID deaths in Black Hispanic infants occurred at one-quarter the rate of non-Hispanic Black infants, (0.21 vs 0.87 deaths per 1000 births), Black Hispanic infants nonetheless experienced a SUID rate exceeding that of any other race among Hispanic infants. Although the Black Hispanic SUID rates approximated US average and non-Hispanic White SUID rates, this comparison must also consider the marked increase from SUID rates in infants of non–US-born Black Hispanic mothers. Our research reinforces the well-established concern in SUID research about the disparate impact of race. It also adds a Hispanic corollary, which illustrates some protection for Black Hispanic infants, but not enough to overcome the underlying dynamic of race-associated disparities.

Our findings highlight several aspects of the Hispanic paradox. First, the overall lower SUID rates in infants born to non–US-born Hispanic mothers compared with the US SUID rate suggests a healthy migrant effect,^28^ which is considered to reflect the fact that undertaking emigration requires a higher level of health that is reflected in medical outcomes.^29^ As SUID correlates with pregnancy outcomes and shares certain etiologic factors with stillbirth or neonatal complications,^30,31,32^ this aligns with research showing that better maternal health translates into lower SUID risk in Hispanic infants, despite adverse SDOH.

In addition, the significantly increased SUID rates observed in infants born to US-born Hispanic mothers (assumed to be later generations) compared with infants born to non–US-born Hispanic mothers (assumed to be first-generation immigrants), aligns with the acculturation paradox, where health outcomes worsen in later immigrant generations despite improvements in health access and personal resources.^14,33^ It has been postulated that there are unrecognized strengths in the non–US-born Hispanic family culture that mitigate risk, such as cultural norms supporting maternity and child-rearing and a greater role for the extended family that may confer protection.^34^ It has been further proposed that these protective norms weaken with acculturation to the US lifestyle, influencing child-rearing practices, limiting extended family involvement, and weakening supports for families with young infants. Indeed, negative health consequences from acculturation can be seen in pregnancy outcomes,^35^ breastfeeding rates,^36^ and maternal smoking,^37^ all of which are potentially relevant to SUID risk.

The notably high SUID rates found in infants born to non–US-born Puerto Rican mothers, markedly higher than rates for infants born to other Hispanic non–US-born mothers, may be a special case of the acculturation effect. Although culturally Hispanic, Puerto Rico has been heavily influenced by the US mainland culture for more than 100 years, and Puerto Rican–born individuals have adopted many aspects of the US mainland lifestyle. This influence likely includes a modifying effect on some of the unrecognized strengths hypothesized to confer a protective factor among Hispanic groups.

Our analysis suggests that US-born generations, who generally achieve less poverty, higher levels of literacy, and greater access to health care, have higher SUID risk. The approach to SUID prevention in the US relies on a strategy of public awareness and clinician counseling during obstetric and pediatric health care encounters, and a view that SUID risk is rooted in health-related decisions in prenatal and child-rearing practices that will benefit from preventive counseling and care. Although this would seem to predict generational improvements in SUID outcomes, we instead found the opposite. Our data raise questions about limitations of this approach in the US, and our findings challenge us to ask why. The rationale for greater diversity and inclusion in medical research includes a desire to address both inaccuracies in how research may pertain to underrepresented minoritized groups, as well as factual misrepresentations about the certainty of conclusions.^38^ Our results suggest that both issues may be at play. Work remains to be done in parsing out the role of SDOH and systemic disparities in SUID risk.

### Limitations

Our study has several limitations. Although our population-level dataset is large, it relies on self-reported data, and underreporting is a concern with Hispanic health data.^39^ Perceived biases, unclear racial categories, and complex family histories may all affect the reliability of these self-reported data. Also, while stringent in our representation of missing data, there was a substantial amount of missing race, ethnicity, and maternal birth country data. Data from Puerto Rico were also limited and difficult to extract from mainland data, which may influence results. Paternal factors were not included in the analysis. Indexing data to maternal reports of birth location is an imprecise indicator of generational and/or acculturation effects, and complex issues related to minoritization were not examined. We have also devoted little attention to other non–US-born populations with similarly lowered SUID rates, although we propose this as a focus for future research.

## Conclusions

In this cohort study of SUID in infants born from 1996 to 2017, we found that US infants born to Hispanic mothers had markedly lower rates and seemingly distinct mechanisms of SUID risk compared with infants born to non-Hispanic mothers. Known SUID risk factors seemed to operate differently in Hispanic infants, with implications for our current understanding of SUID risk factors and SDOH, while also suggesting unidentified protective factors in the Hispanic culture. Understanding how risk operates in this important US population may have implications across all ethnic groups and help us better understand and address the stubborn mortality burden of SUID.

## Notes

Supplement 1. **eTable 1.** Definitions of covariates **eTable 2.** Counts and SUID rates from 1996 to 2017 for the paternal factors according to maternal Hispanic vs non-Hispanic ethnicity. **eTable 3.** Logistic regression model to assess the relationship between SUID and mother’s Hispanic ethnicity **eFigure.** SUID rates over time for Black and White racial subgroups, Hispanic and non-Hispanic

Supplement 2.
Data Sharing Statement
