# Supplementary material for: Sudden Unexpected Infant Death Rates and Social Determinants of Health Among Hispanic Infants
Source: JAMA Netw Open. 2025 Jun 16;8(6):e2515986. doi: 10.1001/jamanetworkopen.2025.15986 (PMC12171938; doi:10.1001/jamanetworkopen.2025.15986)
Supplement: Supplement 1. — eTable 1. Definitions of covariates eTable 2. Counts and SUID rates from 1996 to 2017 for the paternal factors according to maternal Hispanic vs non-Hispanic ethnicity. eTable 3. Logistic regression model to assess the relationship between SUID and mother’s Hispanic ethnicity eFigure. SUID rates over time for Black and White racial subgroups, Hispanic and non-Hispanic [file jamanetwopen-e2515986-s001.pdf]

Supplemental Online Content

Quiñones-Pérez B, Cortina C, Sandholm A, Gray KP, Goldstein RD. Sudden unexpected infant death rates and social determinants of health among Hispanic infants. *JAMA Netw Open*. 2025;8(6):e2515986.
doi:10.1001/jamanetworkopen.2025.15986

eTable 1. Definitions of covariates
eTable 2. Counts and SUID rates from 1996 to 2017 for the paternal factors according to maternal Hispanic vs non-Hispanic ethnicity
eTable 3. Logistic regression model to assess the relationship between SUID and mother's Hispanic ethnicity
eFigure. SUID rates over time for Black and White racial subgroups, Hispanic and non-Hispanic

This supplemental material has been provided by the authors to give readers additional information about their work.

© 2025 Quiñones-Pérez B et al. *JAMA Network Open*.

eTable 1. Definitions of covariates

Data element	Definition	Data Source	Values
Maternal ethnicity	Self-reported maternal ethnicity	NCHS	Hispanic, non-Hispanic
Maternal race	Self-reported maternal race	NCHS	American Indian & Alaskan Native, Asian & Pacific Islander, Black, White, more than one race
Maternal age	Mother's age at infant's birth	NCHS	<20, 20-24, 25-30, 30-34, 35-39, ≥40
Maternal nativity	Place of birth of the mother<br>*U.S. born*: mother was born in one the 50 states or Washington, DC<br>*Foreign born*: mother was born outside of the 50 states and Washington, DC; includes U.S. territories	NCHS	U.S. born (USB), Foreign born (FB)
Maternal hispanic region of origin	Self-reported region that the mother or mother's family is from. Only for Hispanic mothers	NCHS	Central or South American, Cuban, Mexican, Puerto Rican, Other and Unknown Hispanic, Non-Hispanic
Infant's sex	Infant's sex	NCHS	Male, Female
Infant's gestational age	Gestational age in weeks<br>*Premature*: <37 weeks<br>*Term*: ≥37 weeks	NCHS	Premature, Term
Infant's birth weight	Birth weight in grams<br>*Very low:* <1500 g<br>*Low:* 1500-2500 g<br>*Appropriate:* ≥2500 g	NCHS	Very low, Low, Appropriate
Marital status	Mother's marital status at infant's birth	NCHS	Married, Single
Maternal education	Mother's education level. Before 20XX based on years of education. After 20XX based on education level achieved.<br>*Less than high school:* <11 years of education/no high school diploma<br>*High school or greater:* ≥12 years of education/high school diploma or higher education level	NCHS	Less than high school, High school or greater
Maternal cigarette use	Any cigarette use (current or former) by the mother	NCHS	Yes, No
Late or absent prenatal care	First prenatal appointment occurring after the 27th week or 7th month, or not at all	NCHS	Yes, No

© 2025 Quiñones-Pérez B et al. *JAMA Network Open*.

County-level poverty	Infant's birth county population's income level<br>*High poverty*: ≥20% below the poverty level<br>*Low poverty*: <20% below the poverty level	US Census 2021	High poverty, low poverty
County Rurality	Infant's birth county population living on an urban census tract<br>*Mostly Urban*: ≥50% lives in an urban census tract<br>*Mostly or Completely Rural*: <50% lives in an urban census tract	US Census 2020	Mostly Urban, Mostly or Completely Rural
State SUID Rate Quartiles	Infant's state of birth SUID rate quartiles, based on cumulative SUID rates between 1996-2017 in that state<br>*1st Quartile (lowest SUID rates):* CA, CO, CT, HI, MA, MN, NH, NJ, NY, RI, UT, VT*2nd Quartile*: AZ, DC, IA, IL, ME, MI, NE, MN, NV, OR, PA, WA, WI*3rd Quartile:* DE, FL, ID, IN, MD, MO, NC, ND, OH, SD, TX, VA, WY*4th Quartile (highest SUID rates)*: AK, AL, AR, GA, KS, KY, LA, MS, MT, OK, SC, TN, WV	NCHS	1st Quartile, 2nd Quartile, 3rd Quartile, 4th Quartile
Prone Sleeping	*Yes*: Baby sleeping on their stomach either alone or in combination with any other sleeping position.<br>*No*: Baby does not sleep on their stomach at all.	PRAMS	Yes, No
Baby Sleeps with Mother Sometimes or Always	*Yes*: Sometimes or always<br>*No*: Never	PRAMS	Yes, No
Any Maternal Smoking	Answer to question "Did Mom Smoke?"<br>Yes: Mother smoked<br>*No*: Mother did not smoke	PRAMS	Yes, No
Maternal Age Under 20 Years	*Yes*: Mother's age <20 years at infant's birth<br>*No*: Mother's age ≥ 20 years at infant's birth	PRAMS	Yes, No
Unmarried Mother	*Yes*: Mother unmarried at infant's birth<br>*No*: Other	PRAMS	Yes, No
Mother has no High School Degree	*Yes*: <11 years of education<br>*No*: ≥12 years of education	PRAMS	Yes, No
Late or Absent Prenatal Care	*Yes*: First prenatal appointment occurring after the 27th week or 7th month, or not at all<br>*No*: First prenatal appointment occurring before the 27th week or 7th month	PRAMS	Yes, No
Inadequate or Intermediate Kotelchuck Index	A measure of adequacy of prenatal care<br>*Yes*: Inadequate or Intermediate<br>*No*: Adequate or Adequate Plus	PRAMS	Yes, No
Premature Birth	*Yes*: Infant's gestational age < 37 weeks<br>*No*: Infant's gestational age ≥ 37 weeks	PRAMS	Yes, No

© 2025 Quiñones-Pérez B et al. *JAMA Network Open*.

Low Birth Weight	*Yes*: Infant's birth weight < 2500 g<br>*No*: Infant's birth weight ≥ 2500 g	PRAMS	Yes, No
Breastfed	Answer to question "Infant being breast-fed?"<br>Yes: Infant was breastfed<br>*No*: Infant was not breastfed	PRAMS	Yes, No

© 2025 Quiñones-Pérez B et al. *JAMA Network Open*.

eTable 2. Counts and SUID rates from 1996 to 2017 for the paternal factors according to maternal Hispanic vs non-Hispanic ethnicity

	Total, n (%) (N=88,067,608)	Maternal Hispanic Ethnicity, n (%)			Maternal Non-Hispanic Ethnicity, n (%)		
		No SUID (N=19,879,983)	SUID (N=7,173)	SUID Rate[1] (0.36)	No SUID (N=68,132,797)	SUID (N=47,655)	SUID Rate[1] (0.70)
**Paternal Characteristics**							
Paternal Race							
American Indian & Alaskan Native	485,476 (0.6)	47,938 (0.2)	28 (0.4)	0.59	437,026 (0.6)	483 (1.0)	1.1
Asian & Pacific Islander	2,281,347 (2.6)	87,046 (0.4)	29 (0.4)	0.34	2,193,810 (3.2)	462 (1.0)	0.21
Black	7,064,124 (8.0)	506,338 (2.5)	286 (4.0)	0.56	6,551,370 (9.6)	6,130 (12.9)	0.93
White	41,174,887 (46.8)	9,747,589 (49.0)	2,700 (37.6)	0.28	31,410,403 (46.1)	14,195 (29.8)	0.45
More than one race	294,297 (0.3)	61,296 (0.3)	0 (0.0)		233,001 (0.3)	0 (0.0)	
Unknown	36,767,477 (41.7)	9,429,776 (47.4)	4,129 (57.6)		27,307,187 (40.1)	26,385 (55.4)	
Paternal Age (years)							
< 20	2,396,041 (2.7)	827,096 (4.2)	593 (8.3)	0.72	1,566,066 (2.3)	2,285 (4.8)	1.46
20 - 24	11,279,586 (12.8)	3,392,832 (17.1)	1,560 (21.7)	0.46	7,876,465 (11.6)	8,729 (18.3)	1.11
25 - 29	17,786,391 (20.2)	4,357,790 (21.9)	1,287 (17.9)	0.3	13,419,600 (19.7)	7,714 (16.2)	0.57
30 - 34	18,938,714 (21.5)	3,628,661 (18.3)	697 (9.7)	0.19	15,304,248 (22.5)	5,108 (10.7)	0.33
35 - 39	11,870,061 (13.5)	2,109,158 (10.6)	352 (4.9)	0.17	9,757,751 (14.3)	2,800 (5.9)	0.29
≥ 40	17,742,202 (20.1)	3,687,500 (18.5)	2,010 (28.0)	0.54	14,036,010 (20.6)	16,682 (35.0)	1.19
Unknown	8,054,614 (9.1)	1,876,946 (9.4)	674 (9.4)		6,172,657 (9.1)	4,337 (9.1)	
Paternal Hispanic Region of Origin							
Non-Hispanic	58,644,512 (66.6)	2,658,326 (13.4)	1,319 (18.4)	0.5	55,954,340 (82.1)	30,527 (64.1)	0.55
Mexican	11,709,635 (13.3)	10,208,330 (51.3)	2,846 (39.7)	0.28	1,497,403 (2.2)	1,056 (2.2)	0.71
Puerto Rican	1,163,034 (1.3)	752,597 (3.8)	330 (4.6)	0.44	409,839 (0.6)	267 (0.6)	0.65
Cuban	354,330 (0.4)	278,787 (1.4)	36 (0.5)	0.13	75,482 (0.1)	25 (0.1)	0.33
Central or South American	2,397,095 (2.7)	2,067,231 (10.4)	429 (6.0)	0.21	329,309 (0.5)	126 (0.3)	0.38

© 2025 Quiñones-Pérez B et al. *JAMA Network Open*.

Other and unknown Hispanic	1,668,363 (1.9)	1,275,399 (6.4)	388 (5.4)	0.3	392,372 (0.6)	204 (0.4)	0.52
Unknown	12,130,640 (13.8)	2,639,313 (13.3)	1,826 (25.5)		9,474,052 (13.9)	15,449 (32.4)	

© 2025 Quiñones-Pérez B et al. *JAMA Network Open*.

eTable 3. Logistic regression model to assess the relationship between SUID and mother's Hispanic ethnicity

	**SUID Odds Ratio (OR) Hispanics vs non-Hispanic**		**SUID Cases**
	**Univariate OR (95% CI)**	**Adjusted OR (95% CI)**	
Study cohort=88,067,086			
Hispanic Mother (n=19,887,156)	0.52 (0.50 - 0.53)	0.69 (0.67 - 0.71)	7173
Non-Hispanic Mother (n=65,180,452)	Reference	Reference	47655

© 2025 Quiñones-Pérez B et al. *JAMA Network Open*.

eFigure. SUID Rates Over Time for Black and White Racial Subgroups, Hispanic and Non-Hispanic

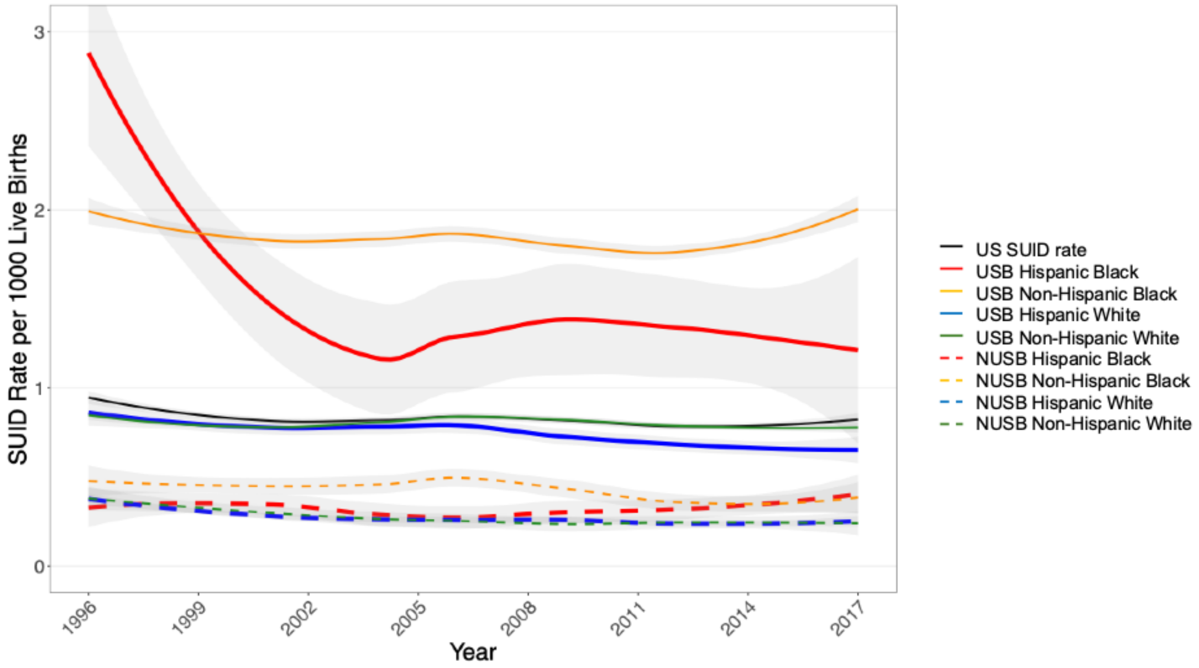

© 2025 Quiñones-Pérez B et al. *JAMA Network Open*.
