# Supplementary material for: Sudden Unexpected Infant Death Rates and Social Determinants of Health Among Hispanic Infants
Source: JAMA Netw Open. 2025 Jun 16;8(6):e2515986. doi: 10.1001/jamanetworkopen.2025.15986 (PMC12171938; doi:10.1001/jamanetworkopen.2025.15986)
Supplement: Supplement 2. — Data Sharing Statement [file jamanetwopen-e2515986-s002.pdf]

Data Sharing Statement

Quiñones-Pérez. Sudden Unexpected Infant Death Rates and Social Determinants of Health Among Hispanic Infants. *JAMA Netw Open*. Published June 16, 2025. doi:10.1001/jamanetworkopen.2025.15986

Data
Data available: Yes
Data types: Data (not involving human participants)
How to access data: NCHS and PRAMS data available through the CDC. Analysis data available by request to richard.goldstein@childrens.harvard.edu
When available: With publication

Supporting Documents
Document types: None

Additional Information
Who can access the data: Anyone requesting data
Types of analyses: For any purpose related to study
Mechanisms of data availability: Without investigator support
